# Lipid-Metabolism-Related Gene Signature Predicts Prognosis and Immune Microenvironment Alterations in Endometrial Cancer

**DOI:** 10.3390/biomedicines13051050

**Published:** 2025-04-26

**Authors:** Zhangxin Wu, Yufei Nie, Deshui Kong, Lixiang Xue, Tianhui He, Kuaile Zhang, Jie Zhang, Chunliang Shang, Hongyan Guo

**Affiliations:** 1 Department of Obstetrics and Gynecology, Peking University Third Hospital, Beijing 100191, China; wzxpku2008@126.com (Z.W.); nyf9055@bjmu.edu.cn (Y.N.);; 2 Institute of Medical Innovation and Research, Peking University Third Hospital, Beijing 100191, China; 3 Biobank, Peking University Third Hospital, Beijing 100191, China; 4 Department of Obstetrics and Gynecology, Beijing Huairou District Traditional Chinese Medicine Hospital, Beijing 101400, China

**Keywords:** endometrial cancer, lipid metabolism, biomarkers, tumor microenvironment, endothelial lipase

## Abstract

**Background/Objectives**: Lipid metabolism plays a crucial role in uterine corpus endometrial carcinoma (UCEC); however, its specific mechanisms remain to be fully elucidated. This study aimed to construct a lipid-metabolism-related prognostic model and explore its association with the tumor immune microenvironment. **Methods**: A total of 552 UCEC and 35 normal tissue samples from The Cancer Genome Atlas (TCGA) database were analyzed to identify differentially expressed lipid-metabolism-related genes (DE-LMRGs). A prognostic risk model was established using univariate Cox analysis, least absolute shrinkage and selection operator (LASSO) regression, and multivariate Cox regression, and its clinical utility was assessed through nomogram construction. Functional enrichment analysis was performed to explore the biological pathways involved. Tumor immune infiltration patterns were evaluated using single-sample Gene Set Enrichment Analysis (ssGSEA), Estimation of Stromal and Immune Cells in Malignant Tumors using Expression Data (ESTIMATE), and Tumor Immune Dysfunction and Exclusion (TIDE) algorithms. Results: Multivariate analysis indicated that the prognostic model had robust predictive value, with AUCs of 0.701, 0.746, and 0.790 for 1-, 3-, and 5-year overall survival predictions. High-risk patients exhibited a suppressed immune microenvironment characterized by reduced immune cell infiltration, lower tumor mutation burden (TMB), and elevated TIDE scores, suggesting potential resistance to immunotherapy. Furthermore, LIPG was identified as a key hub gene through the intersection of nine machine learning algorithms, demonstrating strong associations with both cancer progression and immune infiltration. Functional validation using Cell Counting Kit-8 (CCK-8), wound healing, and transwell migration assays following small interfering RNA (siRNA) transfection demonstrated that LIPG promotes UCEC cell proliferation and migration in vitro. **Conclusions**: These findings highlight the critical role of lipid metabolism in UCEC progression and immune modulation, with LIPG emerging as a potential prognostic biomarker. The identified lipid-metabolism-related gene signature may provide new insights into tumor microenvironment interactions.

## 1. Introduction

Endometrial cancer (EC), the sixth most common cancer type among women worldwide, accounts for approximately 97,000 deaths and 417,000 new cases annually according to the World Health Organization (WHO) [1,2]. The relative 5-year survival rate is approximately 96% for early-stage EC patients, but it drastically declines to about 18% for those with distant metastases [3]. This stark contrast underscores the urgent need for reliable prognostic biomarkers to predict outcomes and guide personalized therapeutic strategies.

Obesity stands out as a major contributor to EC risk, and each 5 kg/m^2^ increase in body mass index (BMI) is linked to a 54% higher risk of cancer. Conversely, successful treatment of obesity significantly reduces cancer risk [4]. Metabolic profiling of cervicovaginal lavage samples has revealed a considerable upregulation of lipids, particularly sphingolipids, fatty acids, and glycerophospholipids, in EC patients compared to benign participants [5]. Furthermore, serum lipid/lipoprotein levels, such as triglycerides, are positively correlated with EC risk, and cholesterol-lowering statin therapy has been shown to improve disease-specific survival in type 2 EC patients [6]. Inhibition of the sterol regulatory element-binding protein (SREBP) pathway by fatostatin has been reported to diminish human EC cell proliferation, trigger cell cycle arrest, and facilitate apoptosis [7]. Collectively, these findings highlight the critical role of lipid metabolism in the development and progression of endometrial cancer.

Within cancer cells, lipid metabolism is reconfigured to enhance lipid oxidation and support energy requirements. Simultaneously, lipid metabolism in immune cells residing in the tumor microenvironment is restructured, exhibiting notable features such as excessive lipid accumulation and upregulation of fatty acid oxidation (FAO). Although this initially enhances cytotoxic function, dysregulation of lipid homeostasis ultimately impairs the anti-tumor immune response [8]. Notably, CD8^+^ tumor-infiltrated lymphocytes (TILs) are vital for anti-tumor defense in EC, yet their infiltration and functionality are suppressed in obese mice and humans [9,10].

Leveraging data from The Cancer Genome Atlas (TCGA), this study pinpointed lipid-metabolism-related genes (LMRGs) with differential expression between EC and normal tissues. A weighted risk model was subsequently developed based on 15 prognostic genes, which was validated through univariate and multivariate analyses. The high-risk group exhibited reduced immune scores, decreased tumor mutational burden (TMB), and elevated tumor immune dysfunction and exclusion (TIDE) indices, collectively indicating an immunosuppressive microenvironment. Furthermore, cellular-level assays confirmed that LIPG, a central node within the protein–protein interaction network, substantially contributes to enhancing the proliferative and migratory capacities of EC cells.

## 2. Materials and Methods

### 2.1. Data Collection and Preprocessing

**Figure 1:**
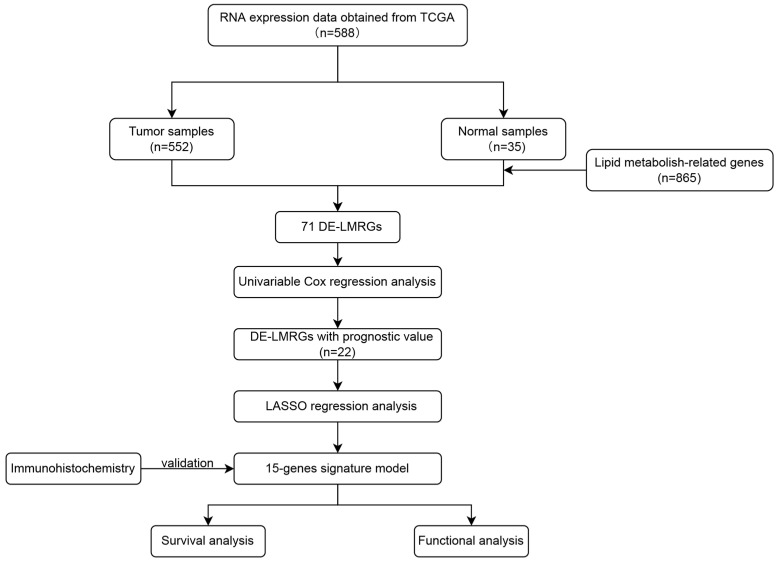
Flowchart of the 15-gene signature model construction. TCGA, The Cancer Genome Atlas; DE-LMRGs, differentially expressed lipid-metabolism-related genes; LASSO regression, least absolute shrinkage and selection operator regression.

RNA sequencing (RNA-seq) expression and clinicopathological data for 552 EC tissue samples and 35 normal tissue samples were obtained from the TCGA UCEC dataset via the Genomic Data Commons (GDC) Data Portal (https://portal.gdc.cancer.gov/, assessed on 1 January 2024). Gene expression data were annotated to map probes to Entrez gene identifiers. LMRGs were curated from four gene sets in the Molecular Signatures Database (MSigDB v7.0): glycerolipid metabolism, phospholipid metabolism, general lipid metabolism, and fatty acid metabolism (Table 1). Figure 1 outlines the workflow from data acquisition to the identification of LMRGs for subsequent analyses.

### 2.2. Construction and Validation of a Lipid Metabolism Risk Model

Expression matrices of LMRGs were obtained, and for genes mapped to multiple probes, the average expression values were computed using the DESeq2 package in R. To uncover candidate LMRGs potentially implicated in EC pathogenesis, differentially expressed LMRGs (DE-LMRGs) between malignant and normal endometrial tissues were identified, using a threshold of adjusted false discovery rate (FDR) < 0.05 and absolute log_2_ fold change (|log_2_FC|) > 2.

To further refine genes with prognostic significance, univariate Cox proportional hazards regression was conducted, resulting in 22 gene candidates. Their mutation landscapes were visualized through OncoPrint plots generated via the cBioportal platform, while their interaction architecture was elucidated by constructing a protein–protein interaction (PPI) network using the String Database (http://string-db.org, assessed on 1 January 2024). In order to mitigate model overfitting, least absolute shrinkage and selection operator (LASSO) regression was implemented with 1000 permutations via the “glmnet” package in R, ultimately identifying a 15-gene LMRG signature.

A prognostic risk model was then formulated by integrating normalized gene expression values (xi) and their corresponding regression coefficients (Coefi) using the following equation: Risk score = ∑(Coefi × xi).

To assess the predictive performance of this model, Kaplan–Meier curves and log-rank tests were applied using the median score as the stratification threshold. Additionally, the area under the ROC curve (AUC) was computed with the “survivalROC” R package. Principal component analysis (PCA) was performed to visualize group separation.

### 2.3. Prognostic Significance and Nomogram Development

To assess the prognostic significance of the constructed risk model in conjunction with clinical variables, both univariate and multivariate Cox proportional hazards analyses were performed, leading to the identification of independent indicators of patient survival. A nomogram integrating these predictors was subsequently established, and its predictive accuracy was verified through calibration curves generated using the “rms” R package.

### 2.4. Functional and Pathway Enrichment Analysis

Differential expression analysis between high- and low-risk patient cohorts was performed under the criteria of |log_2_FC| > 2 and an FDR of <0.05. To explore the functional divergence between the groups, Gene Ontology (GO) and Kyoto Encyclopedia of Genes and Genomes (KEGG) pathway enrichment analyses were carried out utilizing the “clusterProfiler” R package. In addition, Gene Set Enrichment Analysis (GSEA) was subsequently employed to identify survival-related signaling pathways, considering statistical thresholds of *p* < 0.05 and FDR < 0.25.

To identify robust prognostic biomarkers, nine distinct machine learning models, including Elastic Net, GBM (Gradient Boosting Machine), GLM (Generalized Linear Model), KNN (K-Nearest Neighbors), Logit (Logistic Regression), PLS (Partial Least Squares Regression), RF (Random Forest), stepLDA (Stepwise Linear Discriminant Analysis), and SVM (Support Vector Machine), were implemented. The top 10 genes prioritized by each algorithm were cross-compared to detect overlapping candidates.

### 2.5. Tumor Mutational Burden and Immune Infiltration Analysis

TMB was quantified using maftools as mutations per megabase (mut/Mb) according to the formula

$$TMB=\left(\frac{total\;mutant\;bases}{total\;covered\;bases}\right)\times 10^{6}$$

To investigate immune landscape variations between subgroups, a suite of computational tools, including ESTIMATE, ssGSEA (single-sample gene set enrichment analysis), and the TIDE algorithm, was employed.

1. Immune and stromal infiltration levels were inferred using the ESTIMATE algorithm via the “estimate” R package, providing immune and stromal scores for each tumor sample. These scores reflect both the content and spatial composition of tumor-infiltrating immune cells, offering an overview of immune infiltration.
2. The “GSVA” R package was utilized to execute ssGSEA, 24 distinct immune cell populations were profiled within individual tumor samples. Enrichment scores from ssGSEA were compared across risk groups to elucidate immune cell composition patterns related to prognostic stratification.
3. We explored the correlations between the risk score and the levels of immunological checkpoint molecules. These analyses offer insights into the potential role of the risk model in guiding immune checkpoint blockade (ICB) therapies.
4. The TIDE algorithm was applied to estimate ICB therapy responsiveness by modeling immune evasion. It integrated two key mechanisms: T-cell dysfunction in tumors with high cytotoxic T lymphocyte (CTL) infiltration and T-cell exclusion in tumors with low CTL presence. The resulting TIDE scores provide individualized predictions of immunotherapeutic efficacy.

### 2.6. Validation of LMRG Protein Expression Using HPA

To validate the protein expression levels of LMRGs identified in this study, data from the Human Protein Atlas (HPA) were employed. By cross-referencing gene expression data with available protein expression profiles from the HPA, the translational relevance of identified LMRGs was confirmed. Immunohistochemical findings from the HPA database were pivotal in corroborating the biological significance of these genes, thereby enhancing our understanding of lipid metabolism in EC.

### 2.7. Cell Culture and Patient Sample Collection

Normal endometrial epithelial cells, Ishikawa cells (RRID: CVCL_2529), and HEC-1-B cells (RRID: CVCL_0294) were purchased from Meisen CTCC (Zhejiang, China). Normal endometrial epithelial cells were cultured in complete medium (Meisen CTCC-008-0014-CM). Ishikawa and HEC-1-B were cultured in DMEM (Gibco, Grand Island, NY, USA) supplemented with 10% fetal bovine serum (Biological Industries, Kibbutz Beit Ha’emek, Israel) and 1% penicillin/streptomycin (Gibco, Grand Island, NY, USA) under standard conditions (37 °C, 5% CO_2_).

Tumors and paired adjacent normal tissues were collected from 11 EC patients at Peking University Third Hospital (Beijing, China). Written informed consent was obtained from all participants, and the study was approved by the ethics committee of Peking University Third Hospital.

### 2.8. Real-Time PCR (RT-PCR) Analysis

Total RNA was extracted using an RNAeasy kit (Tiangen, Beijing, China) followed by reverse transcription using a reverse transcription kit and SYBR qPCR master mix (Tiangen, Beijing, China). GAPDH was used as an internal control, and relative mRNA expression levels were calculated using the 2^−ΔΔCT^ method. Primer sequences are provided in Table S1.

### 2.9. Gene Silencing via siRNA

Small interfering RNAs (siRNAs) targeting LIPG were synthesized by Sangon Biotech (Shanghai, China). siRNAs transfections were performed using Hieff Trans^®^ Liposomal Transfection reagent (Yeasen Biotechnology, Shanghai, China) and opti-MEM medium (Gibco, Grand Island, NY, USA). Cells were harvested after 48–72 h post-transfection for downstream experiments. Transfection efficiency was assessed using RT-PCR and Western blotting. The siRNA sequences are listed in Table S2.

### 2.10. Western Blot Analysis

Cells were lysed in RIPA lysis buffer (Applygen, Beijing, China) supplemented with 1% phenylmethanesulfonylfluoride (PMSF) (ABclonal, Wuhan, China). Protein concentrations were quantified using a BCA kit (Thermo Fisher Scientific, Waltham, MA, USA). Equal protein amounts (20 μg) were separated via 10% SDS-PAGE and transferred to PVDF membranes (Millipore, Burlington, MA, USA). Membranes were blocked with 5% non-fat milk for 1 h, incubated overnight at 4 °C with primary antibodies (Table S3) and then with HRP-conjugated secondary antibodies for 1.5 h. Proteins were visualized using ECL reagents (NCM Biotech, Suzhou, China).

### 2.11. Cell Viability and Migration Assays

1. CCK-8 Viability Assay: Transfected Ishikawa and HEC-1-B cells were seeded in 96-well plates (2 × 10^3^ cells/well). After incubation with 10% CCK-8 solution (Applygen, Beijing, China) for 1 h, the absorbance at 462 nm was measured using a microplate reader (BioTek Instruments, Winooski, VT, USA).
2. Wound Healing Assay: Confluent monolayers of transfected cells were scratched with a sterile pipette tip, and wound closure was monitored and photographed at 0 and 24 h.
3. Transwell Migration Assay: Transfected cells (3 × 10^4^) were seeded in the upper chambers of transwell inserts (8.0-μm, Corning, Shanghai, China) with serum-free medium, while the lower chambers contained DMEM with 10% fetal bovine serum. After 16 h of incubation, migrated cells were fixed, stained with crystal violet, and visualized under a microscope.

### 2.12. Immune-Related Analysis of LIPG Expression

We performed Spearman correlation analysis to evaluate the expression of LIPG in relation to various immune-infiltrating cells in UCEC. Immune infiltration data for all TCGA samples were obtained from the TIMER2.0 database to ensure consistency and accuracy.

Correlation coefficients were calculated using multiple algorithms and visualized as a heatmap, providing a comprehensive overview of the relationships between LIPG expression and immune cell types across different computational methods.

### 2.13. Statistical Analysis

All statistical analyses were conducted using the R software (v3.6.2) and GraphPad Prism 9. Gene expression differences between groups were evaluated using Student’s *t*-test, while categorical variables were assessed with the chi-square test. Ranked data were analyzed using the Wilcoxon rank-sum test, and comparisons across multiple groups were made using one-way ANOVA. The Benjamini–Hochberg (BH) method was applied to control the FDR for multiple testing corrections using a statistical level of *p* < 0.05.

## 3. Results

### 3.1. Identification of Prognostic LMRGs in UCEC

**Figure 2:**
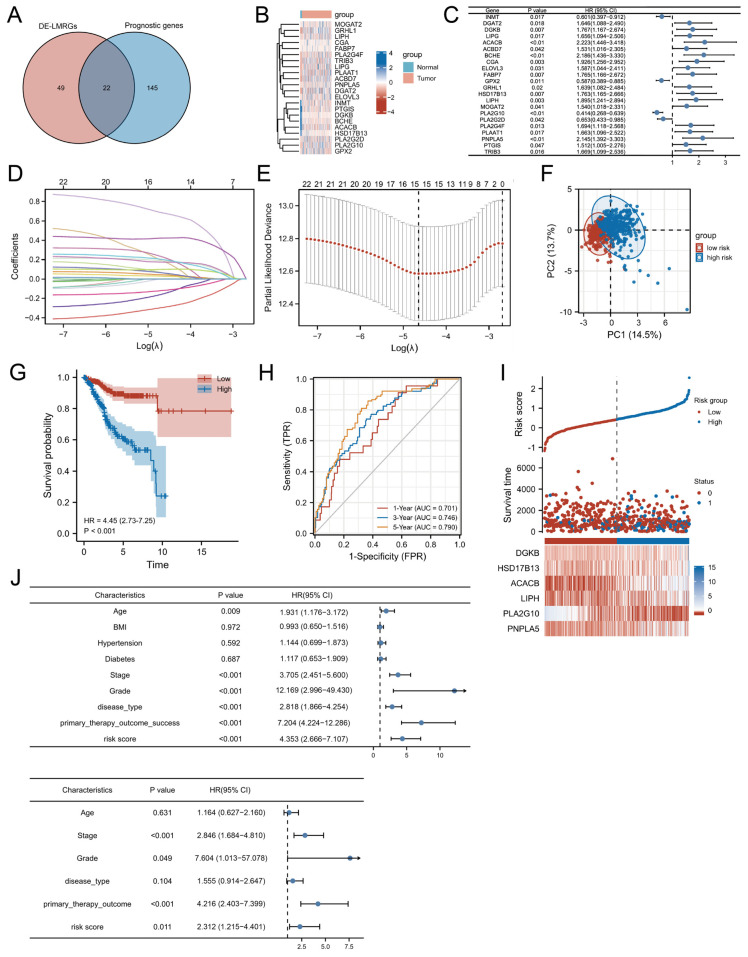
The development of a prognostic LMRG signature. (**A**) Venn diagram depicting the prognostic DEGs. (**B**) Heatmap of the 22 prognostic DEGs, with red representing upregulation and blue indicating downregulation. (**C**) Forest plot showing HRs derived from the univariate Cox regression analysis with gene expression levels as variables. (**D**) LASSO coefficient profiles of the 22 genes in the EC samples. (**E**) Partial likelihood deviance of the LASSO model across different penalty parameter (λ) values. The solid line represents the mean, the shaded band represents ±1 standard error (SE), and the dashed vertical line indicates the λ value at which the model error is minimized. (**F**) Principal component analysis plot. (**G**) Kaplan–Meier survival curves illustrating OS in patients from the two risk groups. (**H**) AUC of time-dependent ROC curves verifying the prognostic accuracy of the risk model. (**I**) Distribution and median of the risk scores. (**J**) Univariate and multivariate Cox regression analysis evaluating the associations between risk scores, clinical parameters, and the patient OS. DE-LMRGs, differentially expressed lipid-metabolism-related genes; HR, hazard ratio; CI, confidence interval; TPR, true positive rate; FPR, false positive rate; AUC, area under the curve; BMI, body mass index.

Among 865 LMRGs, 71 were identified as differentially expressed between UCEC and adjacent normal tissues (q-value < 0.05, |log_2_FC| > 2). Further univariate Cox regression analysis revealed 167 LMRGs significantly associated with overall survival (OS). By intersecting these genes, 22 hub genes with prognostic value and differential expression were identified (Figure 2A). A heatmap depicts their expression patterns across samples (Figure 2B), and their prognostic significance is illustrated in Figure 2C.

Using LASSO regression with a one-standard-error margin, 15 key prognostic LMRGs were further selected from the 22 hub genes: *PTGIS*, *DGKB*, *HSD17B13*, *INMT*, *ACACB*, *LIPH*, *PLAAT1*, *TRIB3*, *PLA2G10*, *DGAT2*, *MOGAT2*, *GRHL1*, *PLA2G2D*, *LIPG*, and *PLA2G4F*. These genes were incorporated into a predictive signature specific to UCEC. Figure 2D,E highlight the LASSO regression process, including the tuning parameter selection and coefficient profiles of the 15 genes.

### 3.2. Construction of a Prognostic LMRG Signature

A risk score was calculated for each patient by summing the expression levels of the 15 selected genes, each weighted by its corresponding LASSO-derived coefficients:

Risk score = ∑(Gene expression × Coefficient)

Risk score = (PTGIS expr.) × 0.0622 + (DGKB expr.) × 0.1840 + (HSD17B13 expr.) × 0.6817 + (INMT expr.) × −0.1530 + (ACACB expr.) × 0.4221 + (LIPH expr.) × 0.1873 + (PLAAT1 expr.) × 0.0291 + (TRIB3 expr.) × 0.0428 + (PLA2G10 expr.) × −0.2927 + (DGAT2 expr.) × 0.1740 + (MOGAT2 expr.) × 0.2245 + (GRHL1 expr.) × 0.0623 + (PLA2G2D expr.) × −0.1179 + (LIPG expr.) × 0.0991 + (PLA2G4F expr.) × 0.2004

PCA revealed distinct clustering of high- and low-risk groups, supporting the robustness of the signature (Figure 2F). Figure 2I illustrates the distribution of risk scores in the TCGA dataset, showing an inverse correlation with overall survival, where higher risk scores are associated with shorter survival times and increased mortality. Notably, patients categorized in the high-risk group succumbed to the disease at a significantly younger age than those in the low-risk group.

Kaplan–Meier survival analysis revealed a markedly poorer survival outcome in the high-risk group (Figure 2G). Time-dependent ROC curve analysis yielded AUC values of 0.701, 0.746, and 0.790 for 1-, 3-, and 5-year OS predictions, respectively, indicating the prognostic model’s potential for long-term survival estimation (Figure 2H).

Our study cohort comprised 552 individuals who presented with a diverse array of clinicopathological features. The prognostic relevance of the risk score was assessed alongside various clinicopathological factors, including age, BMI, histological grade, clinical stage, and primary therapeutic outcomes. Univariate Cox regression analysis identified multiple variables that were significantly associated with OS. However, after adjusting for confounding variables in multivariate analysis, the risk score retained its robust prognostic value, independent of clinical stage, histological grade, and therapeutic outcomes.

The results highlight the independent predictive power of the risk model, as further evidenced by the Kaplan–Meier survival plots (Figure 2J) and comprehensive statistical summaries provided in Table 2, highlighting its critical role in prognostic assessment for EC patients.

### 3.3. Development and Validation of an OS Prediction Nomogram

**Figure 3:**
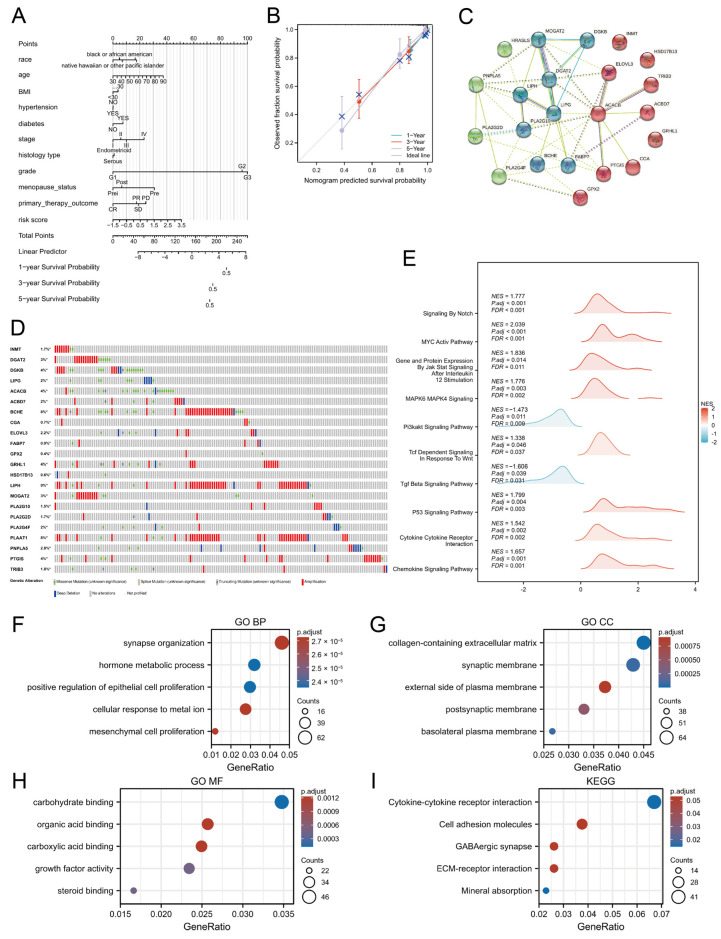
Nomograms and functional enrichment analyses. (**A**) Predictive nomogram estimating 1-, 3-, and 5-year overall survival probabilities in UCEC patients. (**B**) Calibration curves demonstrating the agreement between predicted and observed OS at 1, 3, and 5 years. (**C**) Protein–protein interaction network illustrating interactions among the 22 identified genes. Each node color indicates a cluster determined by k-means clustering. (**D**) Alteration of the 22 candidate genes in clinical samples. (**E**) The enriched biological pathways. (**F**–**H**) Gene Ontology enrichment results showing overrepresented biological processes (BP), cellular components (CC), and molecular functions (MF) among DEGs. (**I**) KEGG pathway analysis of DEGs. BMI, body mass index; NES, normalized enrichment score; FDR, false discovery rate; ECM, extracellular matrix. * *p* < 0.05.

To estimate 1-, 3-, and 5-year overall survival (OS) in patients with uterine corpus endometrial carcinoma (UCEC), a nomogram was constructed by incorporating both clinicopathological variables and the derived risk score. (Figure 3A). Calibration curves showed high concordance between predicted and observed survival probabilities (Figure 3B), underscoring the nomogram’s reliability as a tool for individualized survival prediction.

The nomogram demonstrated high predictive accuracy for overall survival in UCEC patients, as evidenced by the close alignment of the calibration curves with the ideal line, suggesting minimal bias in the survival predictions. This model holds promise as a clinically applicable instrument for generating individualized survival projections, thereby supporting therapeutic decision making and facilitating personalized patient counseling.

### 3.4. Functional Enrichment Analyses of LMRGs

To investigate the interactions among the identified genes, a PPI network was constructed, excluding isolated nodes with no connections. This analysis revealed a densely interconnected network, with LIPG serving as a central hub due to its having the highest number of connections, suggesting its potentially critical role in the pathogenesis of UCEC (Figure 3C).

We also examined mutational data, which revealed amplifications and deletions as the most common mutation types among LMRGs. Notably, *LIPH* exhibited the highest mutation rate, exceeding 4%, which was significantly higher than the six other LMRGs with mutation rates above 4% (Figure 3D).

To elucidate the functional implications of the risk score, enrichment analyses of GO and KEGG pathways were performed using DEGs associated with the prognostic model. GO enrichment revealed significant overrepresentation in biological processes such as synapse organization, collagen-containing extracellular matrix, and carbohydrate-binding functions (Figure 3F–H). KEGG pathway analysis further indicated enrichment in cell adhesion molecules, cytokine–cytokine receptor interactions, and GABAergic synaptic signaling (Figure 3I).

Additionally, GSEA revealed significant correlations between high-risk status and the activation of several pivotal signaling pathways, such as the Notch, Myc, Jak-Stat, Wnt, and P53 pathways (Figure 3E). In contrast, low-risk tissues were enriched in the PI3K and TGF-β pathways.

### 3.5. TMB and Lipid-Metabolism-Related Risk Score

**Figure 4:**
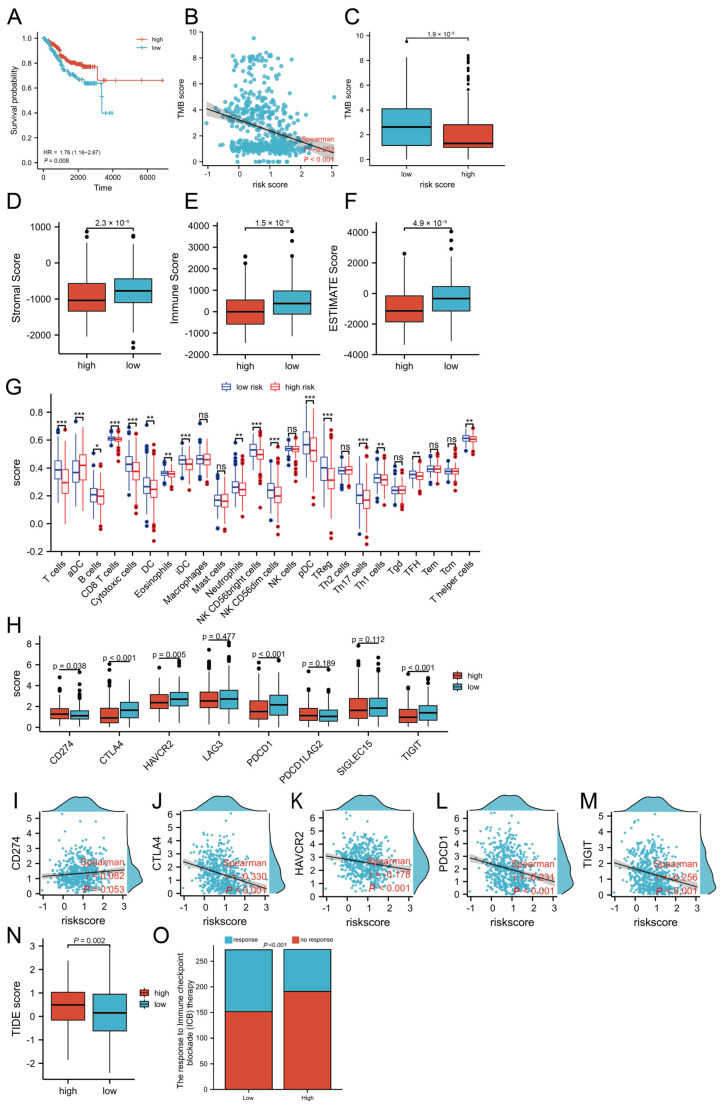
Association of TMB and immune cell infiltration with the risk score. (**A**) The relationship between TMB and patients’ prognostic outcome. (**B**) The correlation of TMB levels with the risk score. (**C**) Lower TMB levels are correlated with the high-risk group (*p* < 0.001). (**D**–**F**) Immunoscore. (**G**) Immune cell infiltration. (**H**) The expression of key immune checkpoint genes. (**I**–**M**) Correlations between immune checkpoint gene expression and the calculated risk score. (**N**,**O**) Response to immunotherapy. TMB, tumor mutational burden; HR, hazard ratio; TIDE, tumor immune dysfunction and exclusion. *p* values are shown as follows: ns, not significant; * *p* < 0.05; ** *p* < 0.01; *** *p* < 0.001.

Recent studies have identified tumor mutational burden (TMB) as a predictive biomarker for immunotherapy response across various malignancies. The lipid-metabolism-related gene signature score may show either a positive or negative correlation with TMB depending on the cancer type [11,12]. In our study, patients with higher TMB demonstrated improved prognosis (Figure 4A). Furthermore, we observed a significant negative correlation between TMB and the lipid-metabolism-related risk score (*p* < 0.001), as shown in Figure 4B. This inverse relationship suggests that elevated TMB levels correspond to lower risk scores, potentially reflecting enhanced lipid homeostasis in EC patients.

Furthermore, individuals classified within the low-risk group displayed significantly elevated TMB levels (*p* < 0.001), as depicted in Figure 4C. This association between TMB and risk score may inform patient stratification for immunotherapeutic interventions and underscores the need for further investigation into the role of TMB in the prognosis and treatment of endometrial cancer.

### 3.6. Connections Between Distinct Immune Cell Infiltration, Immune Checkpoint Genes, and Lipid-Metabolism-Related Risk Score

To investigate the association between LMRGs and the immune landscape in UCEC, we evaluated the immunoscore and immune infiltration levels in relation to the risk score. Our analysis revealed that high-risk patients exhibited significantly lower stromal, immune, and ESTIMATE scores (Figure 4D–F), suggesting an immunosuppressive tumor microenvironment in this subgroup.

To further delineate the immunological landscape in UCEC, we employed ssGSEA to quantify immune cell infiltration. Notably, the low-risk group demonstrated significantly higher enrichment scores for multiple immune cell types, including T cells, B cells, CD8 T cells, cytotoxic cells, dendritic cells (DCs), eosinophils, immature dendritic cells (iDCs), neutrophils, plasmacytoid dendritic cells (pDCs), regulatory T cells (Tregs), Th17 cells, Th1 cells, and follicular helper T (TFH) cells (adjusted *p* < 0.05; Figure 4G). These results indicate that patients in the low-risk group may exhibit a more active and effective anti-tumor immune response.

We next analyzed the expression patterns of several immune checkpoint molecules, including CTLA4 (Cytotoxic T-Lymphocyte-Associated protein 4), HAVCR2 (Human Activating Vascular Cell Receptor 2), PD1 (programmed cell death protein 1), and TIGIT (T cell immunoglobulin and mucin domain containing). High-risk patients exhibited lower expression levels of these genes, while PD-L1 expression was elevated (Figure 4H). Correlation analyses further demonstrated negative associations between the risk score and the expression of *CTLA4*, *HAVCR2*, *PD1*, and *TIGIT*, while a positive correlation was observed between the risk score and *PD-L1* expression (Figure 4I–M).

Furthermore, we utilized the TIDE algorithm to predict immunotherapy response. Low-risk patients had significantly lower TIDE scores, indicating a greater likelihood of benefiting from immune checkpoint blockade (Figure 4N,O). These findings collectively highlight the prognostic and therapeutic implications of lipid-metabolism-related immune dysregulation in UCEC.

### 3.7. Expression of Key Genes in Clinical Samples

**Figure 5:**
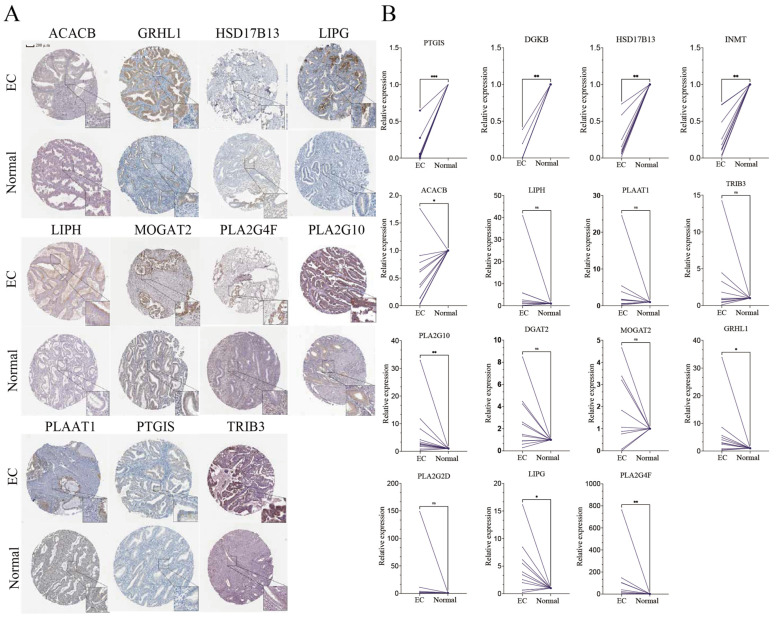
Validation of the LMRGs in EC. (**A**) Representative immunohistochemistry of 11 LMRGs between endometrial cancer and normal tissues in the Human Protein Atlas database. (**B**) Relative mRNA expression levels of LMRGs, normalized to **GAPDH**, in 11 matched pairs of EC and adjacent non-tumorous tissues. EC, endometrial cancer. *p* values are shown as follows: ns, not significant; * *p* < 0.05; ** *p* < 0.01; *** *p* < 0.001.

To validate our findings, expression profiles of the 15 prognostic LMRGs were examined using data from the HPA database. Among these, 11 genes exhibited expression patterns consistent with those observed in the TCGA dataset, thereby reinforcing the reliability of our findings (Figure 5A). However, protein expression data for DGKB, INMT, DGAT2, and PLA2G2D were unavailable in the HPA database.

The transcript levels of the 15 LMRGs were subsequently examined in both EC cell lines and clinical samples. Compared to adjacent normal tissues, tumor tissues exhibited significantly reduced expression of *PTGIS*, *DGKB*, *HSD17B13*, *INMT*, and *ACACB*, while *PLA2G10*, *GRHL1*, *LIPG*, and *PLA2G4F* were markedly upregulated (Figure 5B). In EC cell lines (Ishikawa and HEC-1-B), *INMT* expression was notably lower than in endometrial epithelial cells, whereas *PLAAT1*, *TRIB3*, *GRHL1*, *LIPG*, *PLA2G10*, and *LIPH* were significantly overexpressed (Figure S1).

### 3.8. The Role of LIPG in EC

**Figure 6:**
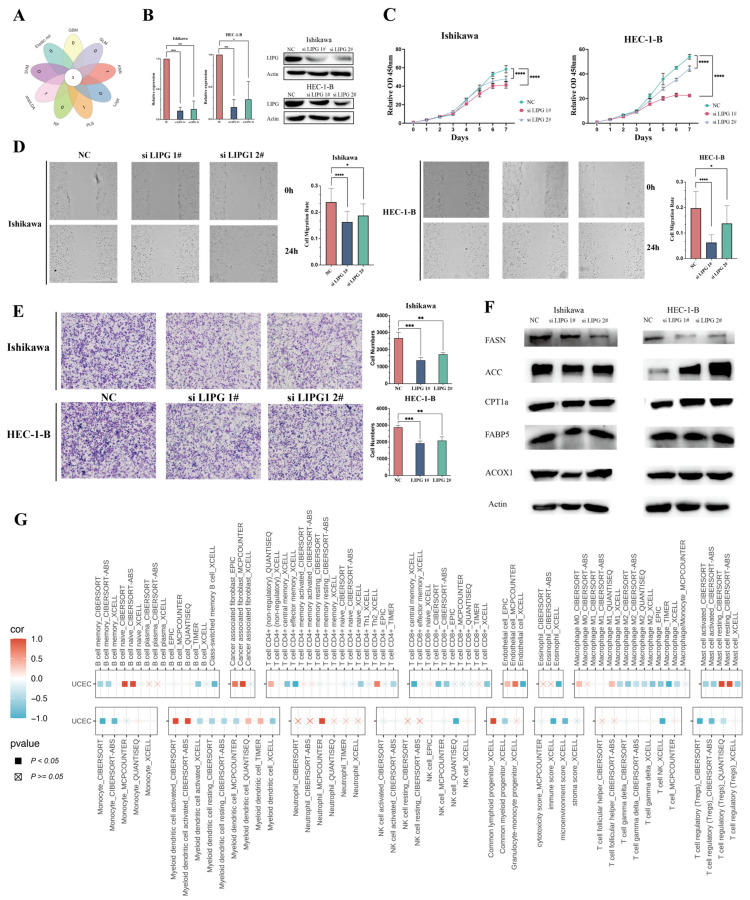
In vitro role of LIPG in endometrial cancer. (**A**) Venn diagram illustrating the overlap of key genes identified by nine distinct machine learning algorithms. (**B**) The effect of LIPG siRNAs in Ishikawa and HEC-1-B cells was evaluated via qRT-PCR and Western blotting. si LIPG #1 and si LIPG #2 denote two distinct siRNA sequences targeting LIPG. (**C**) Proliferative capacities of Ishikawa cells and HEC-1-B cells between the siRNA-transfected group and negative control group were quantified using CCK-8 viability assays. (**D**,**E**) Migrative abilities of Ishikawa and HEC-1-B cells between the siRNA-transfected group and negative control group were assessed using wound healing assays and transwell migration assays, respectively, at 10× magnification. (**F**) Key fatty acid metabolism enzymes in LIPG siRNA-transfected cells compared to control cells were determined using Western blotting. (**G**) Illustration of the correlation between immune infiltrating cells in UCEC and LIPG mRNA expression, as determined by various computational algorithms. FASN, fatty acid synthase; ACC, acetyl-CoA carboxylase; CPT1a, carnitine palmitoyltransferase 1a; FABP5, fatty acid binding protein 5; ACOX1, acyl-CoA oxidase 1; LIPG, endothelial lipase; NC, negative control. *p* values are shown as follows: * *p* < 0.05; ** *p* < 0.01; *** *p* < 0.001; **** *p* < 0.0001.

To identify key prognostic genes among the 22 LMRGs, we applied nine machine learning models, namely, Elastic Net, GBM, GLM, KNN, Logit, PLS, RF, stepLDA, and SVM. Among these LMRGs, *LIPG*, *PTGIS,* and *ACBD7* were consistently identified as key prognostic markers across all nine models (Figure 6A). Notably, *LIPG* exhibited significant upregulation in EC tissues and TCGA datasets, demonstrating a substantial fold change and a correlation with poor prognosis, suggesting its potential role as an oncogene. Additionally, PPI network analysis identified *LIPG* as a central hub with the highest degree of connectivity, further emphasizing its functional significance in EC. Based on these findings, we next explored the functional significance of *LIPG* in EC cell lines.

To investigate the potential oncogenic function of LIPG, we silenced its expression in EC cell lines (Ishikawa and HEC-1-B) using specific siRNAs. Successful knockdown of LIPG was confirmed using qRT-PCR and Western blotting analysis (Figure 6B). LIPG suppression significantly inhibited cell proliferation (Figure 6C) and attenuated migration capacity, as evidenced by wound healing assays (Figure 6D) and transwell migration assays (Figure 6E). These results imply a contributory role for LIPG in promoting EC progression.

LIPG, a crucial lipase involved in lipid and lipoprotein metabolism, predominantly catalyzes the conversion of phospholipids within high-density lipoprotein (HDL) into lysophosphatidylcholine and free fatty acids [13,14]. Upregulation of LIPG in tumor cells has been shown to elevate fatty acid levels and promote lipid storage, supporting cell proliferation [15,16,17]. Consistent with this, LIPG knockdown led to a marked decrease in FASN protein levels in both Ishikawa and HEC-1-B cell lines (Figure 6F), implicating LIPG in fatty acid metabolism regulation.

To examine the association between LIPG expression and the immune landscape in EC, we analyzed immune molecules and cells in EC patients by LIPG expression levels. Multi-algorithm analyses identified an inverse correlation between LIPG expression and the infiltration levels of CD8⁺ T cells, macrophages, and regulatory T cells, as well as the overall immune score (Figure 6G), suggesting an immunosuppressive TME in high-LIPG-expressing EC.

## 4. Discussion

Lipids, encompassing fatty acids, sphingolipids, phospholipids, and triglycerides [15], are biologically diverse molecules that are essential for intracellular signaling, energy storage, and cellular structure [16]. Dysregulation of lipid metabolism has been increasingly linked to metabolic disorders and cancer progression [17,18]. Notably, lipid synthesis inhibitors have demonstrated anticancer effects in preclinical and clinical studies [19,20,21]. While the association between lipid metabolism and various cancers has been well documented, its role in UCEC remains underexplored [22,23,24].

This study focused on evaluating the prognostic relevance of DE-LMRGs in UCEC. We identified 71 DE-LMRGs that were significantly altered between UCEC and adjacent normal tissue samples. Using LASSO and multivariate regression analysis, we constructed a 15-gene lipid-metabolism-based risk signature, comprising *PTGIS*, *DGKB*, *HSD17B13*, *INMT*, *ACACB*, *LIPH*, *PLAAT1*, *TRIB3*, *PLA2G10*, *DGAT2*, *MOGAT2*, *GRHL1*, *PLA2G2D*, *LIPG*, and *PLA2G4F*.

Among these genes, TRIB3, a tumor suppressor, modulates the AKT pathway to inhibit UCEC cell growth and promote apoptosis [25]. PTGIS, associated with poor OS in various cancers, may enhance tumor-associated macrophage infiltration and regulatory T cell (Treg) proliferation within the TME [26]. HSD17B13 suppresses the Warburg effect, reducing hepatocellular carcinoma (HCC) tumor growth [27]. ACACB promotes lipogenesis, supporting rapid cancer cell proliferation [28,29]. LIPH enhances breast cancer cell mobility, potentially facilitating metastasis [30]. DGAT2 is critical for tumor metastasis and progression in gastric cancer [31]. GRHL1, a transcriptional repressor, is epigenetically regulated by the chromatin-modifying agent histone deacetylase 3 (HDAC3) in collaboration with the oncogenic protein MYCN [32]. LIPG, implicated in breast cancer, elevates interferon-stimulated genes (ISGs) expression, potentially by enhancing interferon signaling [33].

In our analysis, *PTGIS*, *DGKB*, *HSD17B13*, *INMT*, and *ACACB* exhibited reduced expression in UCEC tissues relative to their normal counterparts, whereas the other ten genes were upregulated. High expression of *INMT*, *PLA2G10*, and *PLA2G2D* was correlated with poor prognosis, whereas the other twelve genes were associated with favorable outcomes, suggesting their complex role in UCEC pathogenesis.

GSEA revealed that the high-risk UCEC patients exhibited activation of the Notch, Myc, Jak-Stat, Wnt, and P53 pathways, while low-risk patients were associated with the PI3K and TGF-β pathways. The Notch pathway regulates cell proliferation, differentiation, and apoptosis, potentially influencing UCEC by regulating the expression of cell cycle proteins (such as cyclin D1) and cell cycle inhibitory proteins (such as p21) [34]. Dysregulation of the Wnt pathway contributes to endometrial hyperplasia, which may proceed to endometrial cancer [35]. Enhanced activity of both the Wnt and JAK–STAT3 pathways has been strongly associated with epithelial–mesenchymal transition (EMT), a fundamental biological process that facilitates tumor progression by promoting invasion, metastasis, and cellular survival [36].

In vitro experiments suggested that LIPG suppression could inhibit the proliferation and migration of EC cells, indicating its role in tumor progression. Tumor cells are able to maintain viability through metabolic reprogramming, and LIPG upregulation has previously been associated with enhanced proliferation in leukemia [14] and breast cancer [37,38,39]. LIPG may facilitate EC tumor cell growth by increasing lipid utilization. Further experimental studies are needed to elucidate the role of LIPG in EC progression.

Lipid metabolism also influences immune system function through various mechanisms, including signal transduction, energy metabolism, membrane structure, and inflammatory responses [37,39,40]. Aberrant lipid metabolism in the TME leads to immunosuppression, affecting the number and function of tumor-associated macrophages, T cells, dendritic cells, and myeloid-derived suppressor cells [41,42,43]. Consistent with this, high-risk UCEC patients exhibited reduced ESTIMATE scores and decreased infiltration of T cells, B cells, CD8^+^ T cells, DC, eosinophils, iDC, neutrophils, pDC, Treg, Th17 cells, Th1 cells, and TFH cells, underscoring the correlation between lipid metabolism and immune suppression in UCEC.

We further explored the predictive value of the lipid-metabolism-based risk signature for immunotherapy and chemotherapy responses. Previous studies have demonstrated that targeting lipid metabolic pathways can potentiate the effectiveness of immune-based treatments, including anti-PD-1/PD-L1 blockade and adoptive T cell therapy [43,44]. In our study, low-risk patients exhibited a lower expression of immune checkpoint proteins (CTLA4, HAVCR2, PD1, and TIGIT) and a more favorable response to immunotherapy compared to high-risk patients, suggesting that lipid metabolism may influence immunotherapy outcomes in UCEC.

Compared with a previous study that also investigated lipid-metabolism-related gene signatures in EC [45], our study differs in several key aspects. We incorporated a broader set of 1159 LMRGs from multiple MSigDB gene sets (HALLMARK, KEGG, and Reactome), applied more stringent criteria for DEG identification (|log₂FC| > 2), and combined machine learning with PPI network analysis to identify LIPG as a hub gene. Furthermore, we validated its biological role through in vitro experiments, providing deeper insights beyond prognostic modeling. Although the specific DE-LMRGs included in the risk models differed, both studies observed that the low-risk group exhibited significantly higher TMB and potentially greater sensitivity to immunotherapy. This finding emphasizes the importance of lipid metabolism reprogramming in modulating anti-tumor immune responses and warrants further investigation.

Our study has several limitations. Firstly, our analysis was confined to LMRGs and did not encompass a broader spectrum of cancer-related genes, which could offer additional insights into UCEC biology. Secondly, the findings are based on bioinformatic analysis of publicly available datasets. Experimental validation and large-scale clinical studies are needed to confirm the prognostic and therapeutic relevance of the identified risk signature in UCEC.

## 5. Conclusions

The present study concentrated on the analysis of 15 LMRGs to construct a prognostic risk score derived from their gene expression profiles. This risk score was designed to prognosticate the clinical outcomes of patients with UCEC, demonstrating accuracy in outcome prediction. Notably, patients classified with a low-risk profile according to the developed score exhibited more favorable prognoses. Furthermore, LIPG may contribute to the advancement of EC, with its high expression potentially reflecting an immunosuppressive TME in EC. Future research should prioritize experimental and clinical validation of this risk signature to confirm its predictive power and ensure its applicability across diverse patient populations.

## Figures and Tables

**Table 1:** Pathways related to lipid metabolism.

Pathways	Database	Gene Count
HALLMARK_FATTY_ACID_METABOLISM	HALLMARK	158
KEGG_GLYCEROLIPID_METABOLISM	KEGG	49
REACTOME_METABOLISM_OF_LIPIDS	Reactome	741
REACTOME_PHOSPHOLIPID_METABOLISM	Reactome	211
Sum	1159 (unique: 865)	

**Table 2:** Univariable and multivariable analyses for each clinical feature.

Clinical Feature	Number	Univariate Analysis			Multivariate Analysis		
		HR	95% CI	*p* Value	HR	95% CI	*p* Value
DE-LMG Risk Parameter (high-risk/low-risk)	271/273	4.353	2.666–7.107	<0.001	2.312	1.215–4.401	0.011
Age (≥60/<60)	364/178	1.931	1.176–3.172	0.009	1.164	0.627–2.160	0.631
BMI (≥30/<30)	304/208	0.993	0.650–1.516	0.972			
Hypertension (YES/NO)	232/161	1.144	0.699–1.873	0.592			
Diabetes (YES/NO)	100/267	1.117	0.653–1.909	0.687			
Stage (III–IV/I–II)	155/389	3.705	2.451–5.600	<0.001	2.846	1.684–4.810	<0.001
Grade (G2.G3/G1)	446/98	12.169	2.996–49.4320	<0.001	7.604	1.013–57.078	0.049
Disease type (Serous/Endometrioid)	141/401	2.818	1.866–4.254	<0.001	1.555	0.914–2.647	0.104
Primary therapy outcome (CR/not CR)	386/33	7.204	4.224–12.286	<0.001	4.216	2.403–7.399	<0.001

## Data Availability

The raw data supporting the conclusions of this article will be made available by the authors upon request.

## Supplementary Materials

The following supporting information can be downloaded at: https://www.mdpi.com/article/10.3390/biomedicines13051050/s1, Table S1: Primer sequences; Table S2: LIPG siRNA sequences; Table S3: Antibody lists; Figure S1: The relative expression levels of lipid metabolism-related genes compared with GAPDH between EC cells and normal endometrial epithelial cells.

## Abbreviations

The following abbreviations are used in this manuscript:

BMI	body mass index
CTLA4	cytotoxic T-lymphocyte-associated protein 4
CYT	cytolytic activity
DC	dendritic cells
DEGs	differentially expressed genes
DE-LMRGs	differentially expressed lipid-metabolism-associated genes
EaSIeR	Estimate Systems Immune Response
EC	endometrial cancer
FDR	false discovery Rate
GDC	Genomic Data Commons
GO	Gene Ontology
GSEA	Gene Set Enrichment Analysis
HAVCR2	human activating vascular cell receptor 2
HPA	Human Protein Atlas
HRD	homologous recombination defects
iDC	immature dendritic cells
IPS	Immune Prognostic Signature
KEGG	Kyoto Encyclopedia of Genes and Genomes
LASSO	least absolute shrinkage and selection operator
LIPG	endothelial lipase
LMRGs	Lipid-metabolism-related genes
MSigDB	Molecular Signatures Database
OS	overall survival
PCA	principal component analysis
PD1	programmed cell death protein 1
pDC	plasmacytoid dendritic cells
PPI	Protein-protein interaction
SREBP	sterol regulatory-element binding protein
ssGSEA	single-sample gene set enrichment analysis
TCGA	the Cancer Genome Atlas
TFH	follicular helper T
TIDE	Tumor Immune Dysfunction and Exclusion
TIGIT	T cell immunoglobulin and mucin domain containing
TIICs	tumor-infiltrating immune cells
TILs	tumor infiltrated lymphocytes
TIP	Tracking Tumor Immunophenotype
TLS	tertiary lymphoid structures
TMB	tumor mutational burden
Treg	regulatory T cells
UCEC	uterine corpus endometrial carcinoma
WHO	World Health Organization
