# Supplementary material for: Lipid-Metabolism-Related Gene Signature Predicts Prognosis and Immune Microenvironment Alterations in Endometrial Cancer
Source: Biomedicines. 2025 Apr 26;13(5):1050. doi: 10.3390/biomedicines13051050 (PMC12109319; doi:10.3390/biomedicines13051050)
Supplement: Supplementary file 1 [file biomedicines-13-01050-s001.zip › biomedicines-3554564-supplementary.pdf]

Table S1. Primer sequences.

Gene	Forward	Reverse
**GAPDH**	GGAGCGAGATCCCTCCAAAAT	GGCTGTTGTCATACTTCTCATGG
**ACTB**	TGGCACCCAGCACAATGAA	CTAAGTCATAGTCCGCCTAGAAGCA
**LIPG**	TACACGGATGCGGTCAATAATA	CATTCCCGAGAGAAAAATCGTC
**ACACB**	GCCTCTGATAACTCAGGGGAG	CCAGTCCCGTTGGCTTGAA
**PLA2G10**	TGTGGGTTGTGTTGGTCCC	GACGCTCTGATTGACGCACT
**DGAT2**	GAATGGGAGTGGCAATGCTAT	CCTCGAAGATCACCTGCTTGT
**PLA2G2D**	AAAGATGCCACGGACTGGTG	CTTCTGGTAGGTGTCCAGGT
**PLA2G4F**	GGCGGGAAACCTACCCATAC	CCACAGTTGCACATAGCAGT
**PLAAT1**	AGAGTGAGATGGCGTTTAATGA	GACGGAACACTTCGATCAAGTC
**MOGAT2**	CAGGTGCTGGACTATATGGAAG	CACAAGGTCAGCATCATCAGAT

Table S2. LIPG siRNA sequences.

	**sense（5'-3'）**	**antisense（5'-3'）**
**1#**	GCCCUUAGAAGACUGCAGUUUTT	AAACUGCAGUCUUCUAAGGGCTT
**2#**	CUUCGGCUUGAGCAUUGGUAUTT	AUACCAAUGCUCAAGCCGAAGTT

Table S3. Antibody lists.

Antibodies	Source	Identifier
Anti-human β-actin	Proteintech	66009-1
Anti-human LIPG	ABclonal	A1891
Anti-human FASN	Proteintech	10624-1
Anti-human FABP5	Proteintech	12348-1
Anti-human ACC	CST	3676S
Anti-human CPT1a	CST	12252S
Anti-human ACOX1	abcam	ab184032

FASN, Fatty Acid Synthase, ACC, Acetyl-CoA Carboxylase, CPT1a, Carnitine Palmitoyltransferase 1a, FABP5, Fatty Acid Binding Protein 5, ACOX1, Acyl-CoA Oxidase 1.

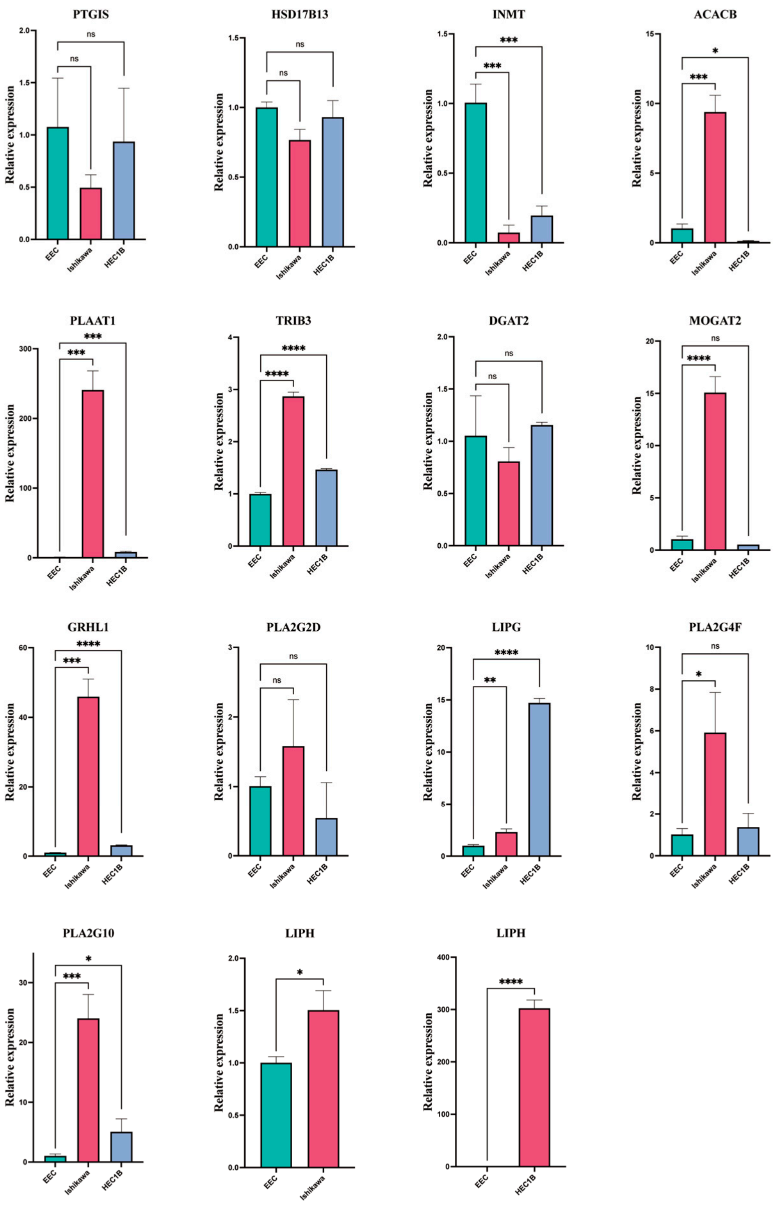

Figure S1. The relative expression levels of lipid metabolism-related genes compared with GAPDH between EC cells and normal endometrial epithelial cells. (*, p< 0.05; **, p< 0.01, ***, p<0.001, ****, p< 0.0001; ns, no significance).
